# Medical Challenges of a Common Variable Immunodeficiency With a TNFRSF13B Gene Mutation in a Simultaneous Kidney and Pancreas Transplant Recipient

**DOI:** 10.7759/cureus.44211

**Published:** 2023-08-27

**Authors:** Miguel T Coimbra, José Silvano, La Salete Martins

**Affiliations:** 1 Nephrology, Hospital Espírito Santo de Évora E.P.E., Évora, PRT; 2 Nephrology, Centro Hospitalar Universitário do Porto, Porto, PRT

**Keywords:** common variable immunodeficiency, chronic kidney disease, calcineurin inhibitors, primary immunodeficiency disorder, pancreas and kidney transplant

## Abstract

Common variable immune deficiency (CVID) is a primary immunodeficiency disorder, with hypogammaglobulinemia and increased susceptibility to recurrent infections, autoimmune disorders, granulomatous diseases and malignancy. Among the solid organ transplant (SOT) recipient population, those with primary immunodeficiency disorders under chronic immunosuppression therapy can theoretically be at higher risk of atypical infections, autoimmune complications and disease recurrence with suboptimal long term graft survival, but literature is scarce. Here, we report a 27-year-old female with type 1 diabetes mellitus, complicated with nephropathy that progressed to end-stage renal disease (ESRD), who had a history of a chronic inflammatory response dysregulation, with chronic monoarthritis, persistent elevation of inflammation markers, recurrent infections, low immunoglobulin G (IgG) and A (IgA) serum levels, a slightly decreased population of memory B cells at flow cytometric immunophenotyping, and a confirmed pathological heterozygous mutation in the tumor necrosis factor receptor superfamily 13B (TNFRSF13B), with a suspected diagnosis of CVID. Whilst on hemodialysis, she received a simultaneous kidney and pancreas transplant from a standard criteria donor (SCD), and our induction and maintenance immunosuppression protocol and prophylaxis regimen allowed for a successful transplant with immediate pancreatic function, with no evidence of renal graft rejection upon biopsy in the early post-transplant period, and no novel episodes of serious infectious complications were recorded during a follow-up period of six months.

## Introduction

Among the subset of people with primary immunodeficiency disorders, common variable immune deficiency (CVID) disorder is one of the most frequent and symptomatic disorders. It is a group of hypogammaglobulinemia syndromes with a poorly understood etiology. There is genetic predisposition to intrinsic B cell and T cell defects, as well as mutations in TNF receptors, but they are not mandatory. Still, tumor necrosis factor receptor superfamily 13B (TNFRSF13B) gene mutations are found in approximately 10% of patients with CVID [1]. Meanwhile, the impact of environmental factors is still uncertain [2]. Clinical presentation, although highly variable, often includes susceptibility to recurrent infections, autoimmune disorders, granulomatous diseases and malignancy [3,4].

Regarding the impact of primary immunodeficiency disorders in renal and pancreatic graft survival and transplant recipient survival under chronic immunosuppression therapy, as well as other complications in the post-transplant period, literature is scarce. In this case report, we aim at discussing the challenges of a patient with diabetic nephropathy and a primary immunodeficiency disorder with a heterozygous mutation in the TNFRSF13B gene, who progressively advanced to end-stage renal disease (ESRD) at an early age and who received a simultaneous renal and pancreas transplant at our institution.

This article was previously presented as a focused oral at the European Renal Association (ERA) Annual Congress in Milan on June 16, 2023.

## Case presentation

A 27-year-old female with type 1 diabetes mellitus, complicated with retinopathy and nephropathy that ultimately progressed to end-stage chronic kidney disease (CKD), had a history of chronic inflammatory response dysregulation. This consisted of chronic left tibiofemoral monoarthritis, persistent elevation of inflammation markers, recurrent infections, low serum levels of immunoglobulin G (IgG) and A (IgA), with the lowest recorded IgG serum level of 638 mg/dL at our institution, and a slightly decreased population of “switched” and “non-switched” memory B cells at flow cytometric immunophenotyping. At the age of 11, she experienced an acute appendicitis and viral meningitis. At 15 years of age, she was diagnosed with acute pneumoniae with pleural effusion. She had several skin and urinary tract infections since childhood, with one episode of acute pyelonephritis with multiple renal abscesses at 19, with no urinary tract malformations, no bladder dysfunction and no previous history of urinary lithiasis. More recently, she was diagnosed with chronic gastritis with positive antiparietal cell antibodies. Immune testing for other auto-antibodies was negative. A diagnosis of common variable immunodeficiency was suspected, and genetic screening for primary immunodeficiency disorders was positive for a heterozygous pathological mutation in the TNFRSF13B gene (c.310T>C (p.Cys104Arg)), and a variant of uncertain significance (VOUS) in the gene of the transcription regulator Broad-complex-tramtrack-bric-a-brac and Cap’n’collar Homology 2 (BACH2) protein. The patient’s family history of chronic oligoarthritis and recurrent infections (from the mother’s side) also hint at a genetic immune deficiency. Monoarthritis symptoms were managed with colchicine, anakinra (interleukin 1-receptor antagonist), with significant improvement, and occasionally, oral and intravenous corticosteroids during exacerbations.

At the age of 24, she experienced a high-risk pregnancy with hemolysis, elevated liver enzymes and low platelets (HELLP) syndrome during the third trimester. Because serum creatinine and non-nephrotic proteinuria progressively worsened since her last pregnancy, a native kidney biopsy was performed, and the results were consistent with diabetic nephropathy, with complement component 3 (C3) and IgG pseudolinear deposits upon microscopic evaluation with immunofluorescence. During clinical follow-up in the post-partum, serum creatinine and non-nephrotic proteinuria progressively worsened, and inflammatory markers were persistently higher from baseline, regarding values of C reactive protein (CRP) of approximately 15 mg/L (>5 mg/L) and erythrocyte sedimentation rate values of approximately 100 mm per hour (> 30 mm per hour), even in the absence of infectious complications, suggesting inflammatory status and auto-immunity. However, monoarthritis symptoms were highly effectively controlled with the introduction of anakinra, and despite being frequently diagnosed with skin and urinary infections on a yearly basis, the last severe infection with need for hospitalization had occurred at the age of 19. Immunoglobulin levels were periodically monitored during our follow-up, and the IgG and IgA deficit was mild to moderate; to our knowledge, Ig supplementation had not previously occurred.

Eventually, kidney disease progression in the last three years led to chronic hemodialysis and, 10 months later, she was accepted at our institution for a simultaneous kidney and pancreas transplant. At the time of transplant, while undergoing hemodialysis, the patient was stable, with no signs of active skin or other type of infection after clinical assessment with physical examination immediately prior to admission for transplant surgery, and no record of urinary infections six months prior. She received a kidney and pancreas transplant from a standard criteria deceased donor, female, aged 27 years old. The donor-receptor pair information is summarized in Table 1. Both donor and receptor had blood group A (ABO Blood Group System) and positive cytomegalovirus (CMV) IgG. Compatibility analysis showed no human leukocyte antigen (HLA) shared antigens, and no HLA antibodies were found. Panel reac tive antibodies (PRA) with complement-dependent cytotoxicity (CDC) test score was 0%, and flow cytometry cross-match (FCXM) was negative. Immunosuppression induction regimen included intravenous anti-thymocyte globulin once daily until day 5 (cumulative dose of 6 mg/kg), oral mycophenolate mofetil (MMF) 1000 mg in a single dose prior to transplant and with dose reduction by half to 500 mg twice daily afterwards, tacrolimus 0.1 mg/kg every 12 hours with adequate trough levels of approximately 10 to 12 ng/mL, and intravenous methylprednisolone pulses for three consecutive days (500 mg, 125 mg and 125mg). Prophylactic infection treatment was standard for simultaneous kidney and pancreas transplant recipients, and included trimethoprim-sulfamethoxazole 480 mg per day (oral), valganciclovir 450 mg every 12 hours (oral), cefotaxime 2 g intravenous (only dose) in the intraoperatory setting and 1 g every eight hours afterwards, intravenous vancomycin 1,000 mg daily, and intravenous fluconazole 100 mg daily. Cefotaxime, vancomycin and fluconazole were suspended after surgical drains removal. Trimethoprim-sulfamethoxazole dose is adjusted according to kidney graft function, and trimethoprim-sulfamethoxazole and valganciclovir prophylaxis were maintained for six months. Thrombosis prophylaxis was adjusted postoperatively to intravenous enoxaparin 20 mg daily and oral acetylsalicylic acid 100 mg daily. IgG and IgA levels were moderately reduced post-transplant, and serum IgG levels remained stable upon discharge at approximately 700-760 mg/dL, which did not mandate for Ig supplementation (> 500 mg/dL) according to our institution. There were no vascular or urologic complications, and, despite a pancreatic cold ischemia time of nearly 12 hours, pancreatic allograft function was optimal. Renal function, however, stabilized with serum creatinine levels of 2.1 mg/dL, with a glomerular filtration rate (GFR) of 33 ml/min/1.73 m² (according to CKD-Epidemiology Collaboration (EPI) 2021 formula).

**Table 1:** Kidney and Pancreas Transplant from a Deceased Donor Clinical characterization and histocompatibility information of the deceased donor and the simultaneous kidney and pancreas graft recipient. CVD: Cardiovascular Disease; HT: Hypertension; ABORh: ABO Blood Group System and Rhesus Blood Group System; PRA-CDC: Panel of Reactive Antibodies with Complement-Derived Cytotoxicity; FCXM: Flow-Cytommetry Crossmatch; HLA: Human Leukocyte Antigen; CMV: Cytomegalovirus; HCV: Hepatitis C Virus; HTLV: Human T-Lymphotropic Virus; Ab: Antibody; Ag: Antigen.

Donor		Recipient		
Identification	27-year-old, female. No prior history of CVD or HT. Serum creatinine at baseline: 0.9 mg/dL	Sensitization events		Pregnancy
		PRA-CDC		0%
		FCXM (B and T cells)		Negative
Cause of death	Hemorrhagic stroke	HLA antibodies	None	
Blood type	ABORh: A+	Blood type	ABORh: A+	
Viral serology tests	CMV: positive IgG anti-CMV; Hepatitis B: negative HBs Ag, anti-HBs Ab and anti-HBc Ab; Hepatitis C: negative anti-HCV Ab; HIV: negative HIV1 and 2 Ab/Ag; HTLV: negative anti-HTLV I/II Ab; Syphilis: negative Treponema Pallidum Ab.	Viral serology tests	CMV: positive IgG anti-CMV; Hepatitis B: negative HBs Ag, anti-HBs Ab and anti-HBc Ab; Hepatitis C: negative anti-HCV Ab; HIV: negative HIV1 and 2 Ab/Ag; HTLV: negative anti-HTLV I/II Ab; Syphilis: negative Treponema Pallidum Ab.	
HLA antigens	HLA class I: HLA-A 01,32; HLA-B 08,64; HLA-C 07,08. HLA class II: HLA-DR 17,07; HLA-DQ 02,-; HLA-DP 03,45.	HLA antigens	HLA class I: HLA-A 02,-; HLA-B 35,44; HLA-C 04,05. HLA class II: HLA-DR 03,04.	
HLA mismatch: 6 in 6 (2 in HLA-A, 2 in HLA-B and 2 in HLA-DR)				

During our six-month follow-up after discharge, we kept a maintenance immunosuppression protocol of tacrolimus (at trough levels between 6 and 8 ng/mL), MMF (500 mg twice daily at half the standard maintenance dose) and prednisolone (20 mg daily initiated at day 3 post-transplant, tapering to 17.5 mg per day at discharge, and inducing a slow taper until the dose of 5 mg of prednisolone is reached by month 6 of maintenance therapy). So far, renal function remained stable, with no serious infectious complications during this time period. Serum IgG levels remained mildly to moderately decreased, without intravenous Immunoglobulin replacement therapy during the follow-up period. Two weeks after discharge, a single urine culture in routine ambulatory assessment detected a Candida *glabrata *(> 10^4^ CFU/mL), which was not present in the following two consecutive urine samples and the patient had no urinary symptoms, and thus we did not provide further treatment. Of note, there was an event of acute gastroenteritis with acute kidney injury (maximum serum creatinine of 2.8 mg/dL) at month 5 post-transplant, which promptly resolved after a five-day course of antibiotics (ciprofloxacin) and hydration. Viral load of CMV and polyomavirus BK (human polyomavirus 1) were both negative. A renal graft biopsy performed upon Hospital admission showed signs of acute tubular necrosis, with minimal interstitial inflammation, not meeting criteria for the diagnosis of acute graft rejection. After a week, serum creatinine returned to baseline levels.

## Discussion

**Figure 1:**
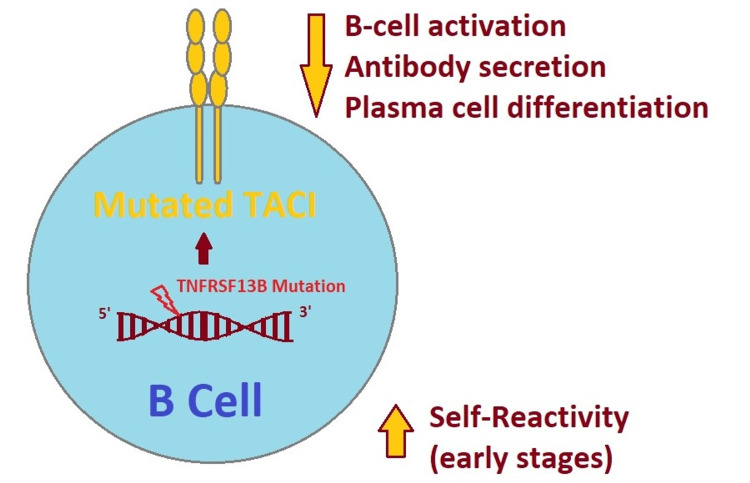
B cell with a mutation in the TNFRSF13B gene In CVID, a TNFRSF13B gene mutation expressed in a B-cell lymphocyte leading to a mutated transmembrane activator, calcium modulator and cyclophilin ligand interactor (TACI) receptor. A mutated TACI receptor can result in decreased B-cell activation, antibody secretion and inhibit differentiation into plasma cells. In early stages of the disease, it can also enhance immune dysregulation by increasing self-reactivity [10].

The TNFRSF13B gene is a member of the TNF receptor superfamily, and a key factor in plasma cell differentiation and production of serum immunoglobulins, and patients with a mutant TNFRSF13B gene are at increased risk of autoimmunity and B cell dysregulation (see Figure 1) [1,5-8]. Although genetic screening reported a VOUS of the patient’s BACH2 gene, it could be pathological, since there have been several BACH2 polymorphisms reported in association with autoimmune diseases, including type 1 diabetes mellitus [9]. CVID is still not fully understood, as it is believed to be a polygenic disease that can be diagnosed by exclusion criteria, and is highly suspected in our case according to patient and family history and laboratory findings, meeting most of the CVID International Consensus (ICON) 2015 criteria [2].

Renal insufficiency occurs in approximately 2% of CVID patients [11,12]. Some studies have demonstrated an increased risk of proteinuria and higher rates of metabolic acidosis in patients with CVID, but without significant differences in GFR when compared to healthy individuals [13]. Other studies showed a disproportionately high incidence of membranous glomerulopathy and tubulointerstitial nephritis in kidney biopsies of patients with CKD and CVID [14]. Also, an hyper-regulated inflammatory state with persistently increased serum inflammatory markers could have accelerated kidney function deterioration, and some authors have found that most patients with CVID and renal dysfunction have immune deposits (IgG or otherwise) upon biopsy and immunofluorescence, although specificity is low, and nearly half of the study population with CVID described by Caza et al. were diagnosed with immune complex glomerulonephritis with capillary loop immune deposits in native kidney biopsy samples [14]. We have hypothesized that an accelerated chronic kidney dysfunction during a time period of three years could have been promoted by any of these factors. However, native kidney biopsy was mainly compatible with renal kidney disease, without any other findings that have been reported to be increased in patients with primary immunodeficiency disorders that could justify accelerated progression of kidney dysfunction, already mentioned above. Although the patient had chronic elevated inflammatory markers, the biopsy was made with patient stability (no serious infections or exacerbation of autoimmune symptoms previous to biopsy). Still, our patient was already chronically treated with anakinra and colchicine, and we did not find enough clinical benefit with chronic systemic corticosteroid treatment in improving renal outcomes in our patient prior to transplant, after weighing for infectious and other risks. Also, in the post-transplant setting, while performing a kidney transplant biopsy due to acute graft dysfunction, we did not find any major inflammatory activity, and we ruled out acute graft rejection.

Regarding the choice of immunosuppression therapy, our protocol was very similar to standard induction protocol for simultaneous kidney and pancreas transplant recipients. Due to a regimen with anti-thymocyte globulin in the simultaneous pancreas and kidney graft recipients, and its consequences in the depletion of T cells, we find it safe to reduce the maintenance dose of MMF to 500 mg every 12 hours in selected cases, after the first administration of anti-thymocyte globulin, at half the dose from standard protocol, since we have found evidence that solid organ transplant (SOT) recipients with immunodeficiency disorders are at high risk of atypical infections [15]. However, the infectious risk should always be weighed against the risk of graft rejection and autoimmune complications. Literature in kidney graft patients with primary immunodeficiency disorders is more scarce, but some case reports refer to acute kidney graft rejection and numerous infections in the early post-transplant period, such as oral candidiasis, infections of the sinopulmonary tract and severe bacteremia, as well as disproportionate immune abnormalities not solely explained by immunosuppression, and a newly diagnosed acquired agammaglobulinemia after kidney transplantation [16,17,18]. Still, in this case, concerning her previous history of chronic and serious infections, we opted for an induction regimen with anti-thymocyte globulin and tacrolimus, with reduced MMF dose, and we followed standard prophylaxis treatment. We also considered belatacept as a possible alternative in maintenance immunosuppressive therapy to tacrolimus, since this is a patient with chronic allograft dysfunction, and as such minimizing calcineurin inhibitor toxicity is a major concern. According to the BENEFIT trial, patients receiving a kidney from a standard criteria deceased donor with belatacept immunosuppression had significantly less risk of graft loss and a notable decrease in the risk of serious infectious events (except for viral infections), compared to Cyclosporine A [19]. However, in other studies, belatacept seems to increase the risk of biopsy-proven acute rejection when compared to tacrolimus, but can be associated with a lower incidence of donor-specific antibodies and improved renal function, albeit these studies are underpowered [20]. While it is still unknown if belatacept can improve post-transplant outcomes compared to tacrolimus in the subset of transplant recipients with primary immunodeficiency disorders, we believe it should be considered as an alternative in patients with serious infectious complications, calcineurin inhibitor (CNI) nephrotoxicity and to allow for prednisolone dose reduction, as long as it does not prohibitively increase the risk of graft rejection.

## Conclusions

With this case report, we wish to further contribute with evidence of renal outcomes and complications in kidney transplant recipients with primary immunodeficiencies, more specifically in the renal and pancreas transplant setting. We are currently monitoring our patient closely, and we will consider immunosuppressive regimen changes should any serious adverse event, infectious or otherwise, arise. Regardless, so far, our case was a success.
